# Brown adipose tissue-derived sEV-lncRNA Morrbid contributes to cold acclimation-induced cardioprotection against extreme cold

**DOI:** 10.1016/j.jlr.2026.101082

**Published:** 2026-06-19

**Authors:** Kai Zhang, Yuwei Hao, Ningxin Li, Bingbing Meng, Chang Liu, Xiaoying Zhang, Jingsheng Lou, Qiang Fu, Yanhong Liu, Jiangbei Cao, Cheng Quan, Weidong Mi, Hao Li

**Affiliations:** 1 Department of Anesthesiology, The First Medical Center, Chinese PLAGH, Beijing, China; 2 Medical School of Chinese People’s Liberation Army General Hospital (PLAGH), Beijing, China; 3 Department of Radiology, Peking Union Medical College Hospital, Chinese Academy of Medical Sciences and Peking Union Medical College, Beijing, China; 4 School of Medicine, Nankai University, Tianjin, China; 5 National Clinical Research Center for Geriatric Diseases, Chinese PLA General Hospital, Beijing, China; 6 State Key Laboratory of Medical Proteomics, National Center for Protein Sciences, Beijing Institute of Radiation Medicine, Beijing, China

**Keywords:** cold acclimation, brown adipose tissue, extracellular vesicles, lncRNA, Morrbid, myocardial injury, Serpine1, extreme cold

## Abstract

Extreme cold causes myocardial injury; however, cold acclimation (CA) enhances myocardial tolerance. This study investigated whether brown adipose tissue (BAT)-derived extracellular vesicles (BAT-EVs) contribute to CA-induced cardioprotection. Mice were subjected to CA or acute cold stress. Cardiac function was assessed using echocardiography and biomarkers. Interscapular BAT (iBAT) ablation has confirmed the necessity of iBAT. EVs were characterized and tested in HL-1 cardiomyocytes. Transcriptomics and specific gene knockdowns have identified key molecular mechanisms. CA preserved cardiac function and integrity under −25°C stress, and these effects were abolished by iBAT ablation. EVs from CA mice mediate this protection by mitigating cardiomyocyte apoptosis. Mechanistically, lncRNA *Morrbid* was enriched in iBAT and serum EVs after CA. iBAT-specific *Morrbid* knockdown significantly attenuates EV-mediated protection. Furthermore, *Morrbid* was associated with upregulation of *Serpine1* in cardiomyocytes; silencing *Serpine1* abolished anti-apoptotic benefits. This study identified a BAT-Heart axis where CA stimulates the release of *Morrbid*-enriched EVs. These vesicles serve as a mechanism to confer remote cardioprotection, potentially by upregulating cardiac *Serpine1* and suppressing apoptosis.

## Graphical abstract

**Figure:**
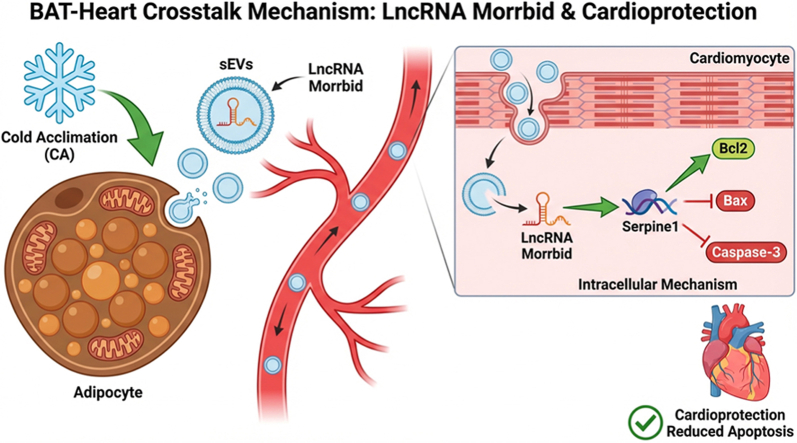

Extreme cold serves as a potent physiological stressor capable of inducing direct myocardial damage and substantially increasing the risk of cardiovascular events, including coronary artery disease and heart failure (1, 2, 3, 4, 5). Cardiovascular events are the leading cause of mortality. Studies have reported that prolonged exposure to cold waves is associated with cumulative excess mortality risks exceeding 28% (6, 7). Despite global warming trends, extreme cold events, such as polar vortex disruptions, occur with increasing frequency and intensity, presenting a severe public health challenge (8, 9). To cope with cold stress, mammals acquire cold acclimation (CA) through repeated exposure to low temperatures, a physiological adaptation that enhances thermogenesis and metabolic health (10). However, its potential as a defense mechanism against environmental cold injury remains largely unexplored.

While CA enhances stress tolerance under various conditions, its potential to directly protect the heart from environmental cold injury remains unexplored. Notably, the mechanisms behind this environmental cold injury are different from myocardial ischemia-reperfusion injury (IRI) (11, 12, 13, 14, 15). Addressing this gap requires identifying the mediators of the systemic effects of CA. As the central effector of CA, brown adipose tissue (BAT) has traditionally been viewed as a thermogenic engine that maintains body temperature (16). However, recent paradigms have redefined BAT as a dynamic endocrine organ capable of crosstalk with distant tissues (17, 18). While soluble “batokines” like FGF21 and NRG4 are known to modulate other organ functions (19, 20, 21), a more sophisticated mode of communication involves extracellular vesicles (EVs) (22, 23). These nano-sized messengers carry complex bioactive cargoes, including proteins, lipids, and non-coding RNAs, facilitating precise intercellular signaling (24, 25, 26, 27, 28). Based on this premise, we hypothesized a novel BAT-Heart axis. In this framework, cold acclimation not only enhances local thermogenesis in BAT but also stimulates the release of specialized EVs acting as long-distance carriers of protective molecular signals. These vesicles traverse the circulation to reach cardiomyocytes, thereby conferring remote resistance against cold-induced injury.

Therefore, this study aims to explore the communication mechanisms between BAT and the heart in extreme cold environments through in vivo and in vitro experiments. Specifically, we aimed to (1) validate the direct cardioprotective efficacy of CA against extreme cold stress, (2) investigate whether this protection is transferable via circulating EVs, with BAT acting as a critical cellular source, and (3) characterize the specific transcriptomic landscape of these vesicles to identify key functional molecules driving this resilience. Through this investigation, we sought to establish a new therapeutic paradigm for the prevention of cold-related cardiovascular injury.

## Materials and methods

### Animals, CA, and extreme cold model

Six-to eight-week-old male C57BL/6J mice of specific-pathogen-free (SPF) grade were obtained from Beijing Yaokang Medical Technology Co., Ltd. Ethical approval was obtained from the Animal Experiment Ethics Committee of the Chinese PLAGH. Mice were maintained under SPF conditions with unrestricted access to food and water. For CA, mice were exposed to 8°C for 21 days in a temperature-controlled chamber, whereas control (Con) groups were maintained at 25°C. To establish the extreme cold model, mice were subjected to −25°C for 2 h/day for 5 days (cold stress, CS). Immediately after each cold exposure session, mice were transferred to a temperature-controlled warming incubator to ensure gradual and stable rewarming, thereby preventing cardiovascular collapse associated with rapid ambient temperature changes. Animals were continuously monitored by trained researchers throughout the stress and recovery phases for signs of excessive suffering, with strict humane endpoints enforced. During the experiment, none of the mice were subjected to any drug intervention, and the CA model was constructed only by regulating ambient temperature.

### Surgical iBAT ablation

The mice were anesthetized using 1.5% isoflurane and placed in a prone position. A 1.5 cm incision was made in the area between the shoulder blades, the interscapular BAT (iBAT) was gently removed, and the incision was closed using interrupted sutures. The sham control group underwent all surgical steps, except iBAT removal. The mice were allowed to recover for 7 days, after which they were assigned to different groups for exposure to extreme cold temperatures.

### In situ adeno-associated virus 9 (AAV9) injection

To achieve iBAT-specific knockdown of *Morrbid*, AAV9 vectors carrying a short hairpin RNA (shRNA) targeting Morrbid or a scrambled control sequence were designed and constructed by Hanbio Biotechnology. The shRNA expression was driven by the U6 promoter. The target sequence for Morrbid was 5′-GCGCATATCTTGGGATCTTTT-3′. A non-targeting scrambled shRNA sequence (showing no homology to any known mouse transcript) was used as the negative control (AAV9-NC). The titer of the AAV9 vectors was 1 × 10^12^ vg/ml. For in situ injection, the mice were anesthetized with 1.5% isoflurane and placed in a prone position on a surgical platform. The fur over the interscapular region was shaved, and the skin was incised to surgically expose iBAT. A total of 16 μl of AAV9-sh*Morrbid* or AAV9-NC viral suspension was injected into all four lobes of iBAT at equal volumes (4 μl per lobe). The incision was then closed with interrupted sutures, and the mice were allowed to recover for 3 weeks to ensure stable gene knockdown before subsequent experiments.

### Establishment of HL-1 mouse cell culture and cold injury model

Murine HL-1 cardiomyocyte-like cells (Catalogue No.: SCC065; Sigma-Aldrich) were cultured in Claycomb medium (Sigma-Aldrich) enriched with 10% fetal bovine serum (Thermo Fisher Scientific), 0.1 mM norepinephrine (Solarbio), and 2 mM L-glutamine (Solarbio). The cells were maintained in a humid environment at 37°C with 5% CO_2_. For the cold injury model, we followed established protocols: HL-1 cells were incubated at 32°C with 5% CO_2_ for 24 h, while all other conditions remained identical to those of the control group (29).

### Echocardiography

Echocardiographic assessments were performed on day 5 of the extreme cold stress protocol, exactly 4 h after the mice were transferred to a warming incubator and had fully resumed normal physical activity. This time point was chosen to evaluate stable cardiac impairment while avoiding acute hemodynamic fluctuations during the early rewarming phase. Mice were anesthetized with 1.5% isoflurane, titrated to achieve light sedation (loss of righting reflex while maintaining regular respiration) to avoid deep anesthesia-induced myocardial depression. Mice were then placed in a supine position. Fur on the thoracic and abdominal regions was shaved on a plate maintained at 37°C. An ultrasound system was used for the ultrasound examination. After obtaining a longitudinal section of the sternum, the probe was rotated 90° clockwise to switch from the long-axis to the short-axis view of the left ventricle. M-mode ultrasonography was performed in the long-axis view. The measurements included the left ventricular internal diameter in diastole (LVIDd), left ventricular internal diameter in systole (LVIDs), left ventricular ejection fraction (LVEF), and fractional shortening (LVFS) of the left ventricle in the short-axis plane.

### Enzyme-linked immunosorbent assay (ELISA)

ELISA was used to detect serum biomarkers. Mice cTnI (Cat. 201–11–0640) and mouse CK-MB (Cat: 201–11–0312) assay kits were obtained from SunRed Biotechnology Company (Shanghai, China). Following the kit guidelines, each parameter was measured using an ELISA reader (CMax Plus; Molecular Devices).

### Histology and immunofluorescence

Tissues were fixed in 4% paraformaldehyde and sectioned after embedding in paraffin. Paraffin-embedded tissues were cut, deparaffinized, hydrated, and stained with hematoxylin and eosin (H&E) or Masson’s trichrome. For immunofluorescence staining, samples were initially permeabilized, blocked with 3% BSA, and incubated overnight at 4°C with UCP1 primary antibodies (Sigma, U6382, 1:100) in a humidified chamber. After washing, the samples were incubated with CY3-conjugated goat anti-rabbit IgG secondary antibodies (1:300 dilution; GB21303; Servicebio) for 50 min at room temperature in the dark, followed by tissue autofluorescence quenching. Nuclei were counterstained with DAPI (G1012, Servicebio). Image acquisition was performed using a fluorescence microscope (Eclipse C1, Nikon, Japan) equipped with a 40×objective. The acquisition settings were strictly controlled using specific excitation (Ex) and emission (Em) wavelengths: DAPI (Ex: 330–380 nm, Em: 420 nm) and CY3 (Ex: 510–560 nm, Em: 590 nm). Digital images were browsed and analyzed using Saiviewer software (Servicebio). All images across different groups were captured under identical acquisition settings to ensure comparability. For HE staining, myocardial damage and cellular infiltration were graded as previously described (30). Quantitative analysis of Masson’s staining was performed as described above (31), and quantitative analysis of the lipid droplet area and UCP1 immunofluorescence density was also performed as described above (32, 33).

### Western blotting

Briefly, proteins were extracted from mouse BAT, serum EVs, myocardium, and HL-1 cells using a protein extraction kit (Beyotime), and their concentrations were measured using a BCA assay kit (Beyotime). Proteins were isolated using sodium dodecyl sulfate polyacrylamide gel electrophoresis and transferred to a 0.22 μm polyvinylidene fluoride membrane that had been treated with methanol. The membrane was blocked with a 5% nonfat milk solution in Tris-buffered saline-Tween 20 for one h at room temperature and then exposed to the primary antibodies for 24 h at 4°C. Protein bands were visualized and analyzed using a chemiluminescent detection system (SCG-W2000, Servicebio) after 1-h of incubation at room temperature with secondary horseradish peroxidase-conjugated antibodies (#BS12478 and #BS13278; Bioworld).

The primary antibodies included: cleaved caspase-3 (#9661, CST), caspase-3 (ab184787, Abcam), Bcl-2 (#15069, CST), Bax (ab32503, Abcam), Serpine1 (ab182973, Abcam), β-actin (#4970, CST), β-Tubulin (#2128S, CST), HSP70 (ab181606, Abcam), calnexin (10427-2-AP, Proteintech), CD9 (ab263019, Abcam), Alix (ab186429, Abcam), and UCP1 (ab234430, Abcam).

### Nanoflow cytometric analysis and flow

NanoFCM (U30; Xiamen Fuliu Biological Technology) was used to measure the size distribution and concentration of sEVs. Apoptosis was measured using an Annexin V and propidium iodide (PI) double-staining kit (#556547, BD Biosciences). Three million cardiomyocytes were seeded in a 6-well plate and incubated overnight before the experimental steps. After incubation with 0.25% trypsin-EDTA solution (SH30042.01; HyClone) for 5 min, the cells were collected, centrifuged at 400 ×*g* for 5 min, washed three times with ice-cold phosphate-buffered saline (PBS), and resuspended at a density of 1 × 10^5^ cells/ml. Cells (500 μl) were double stained with Annexin V and PI, according to the manufacturer’s instructions. Briefly, cells were treated with Annexin V and PI (1:100 v/v) for 10 min in the dark at room temperature. A FACSCalibur cell sorter (Beckman Coulter, Inc) was used for flow cytometry analysis. This test differentiated between intact (Annexin^−^/PI^−^), early apoptotic (Annexin^+^/PI^−^), and late apoptotic (Annexin^+^/PI^+^) cells.

### Cell viability

A CCK-8 kit (Beyotime) was used to assess HL-1 cell viability following treatment. Briefly, HL-1 cells from different treatment groups were incubated, followed by the addition of 100 μl of CCK-8 reagent to each well of a 96-well plate, and then cultured for 2 h. The optical density was measured at 450 nm, and the relative cell survival rate (%) was determined.

### Extraction and purification of sEVs (small EVs)

Blood samples were collected from the mice and, after 1 h, centrifuged at 3,000 ×*g* for 15 min to extract the serum. A size-exclusion chromatography (SEC) column was equilibrated with 10 ml of 0.1 M PBS. The pretreated plasma was then loaded onto the column. Once the sample had completely entered the resin matrix, elution was performed using 0.1 M PBS, and a total of 2 ml of EV-rich fractions (collected as two 1-mL fractions) were harvested. The collected fractions were subsequently transferred to a 100 kDa molecular weight cutoff (MWCO) ultrafiltration device and centrifuged at 4,000 × *g* for approximately 2 min to concentrate to approximately 200 μl. The collected crude sEVs were purified using an exosome purification filter column and centrifuged for further analysis. iBAT-derived EVs were isolated from brown adipose tissue by differential ultracentrifugation. Freshly harvested interscapular BAT was minced and digested with 1% collagenase at 37°C with gentle agitation. Digestion was terminated by adding bovine serum albumin. The tissue suspension was then subjected to sequential centrifugation: 300 × *g* for 10 min at 4°C to remove intact cells; 2,000 × *g* for 10 min at 4°C to remove dead cells; and 10,000 × *g* for 30 min at 4°C to remove cell debris. The resulting supernatant was ultracentrifuged at 100,000 × *g* for 70 min at 4°C to pellet the EVs. The pellet was resuspended in PBS and ultracentrifuged again at 100,000 × *g* for 70 min at 4°C to wash the EVs. The final pellet containing BAT-derived EVs was resuspended in an appropriate volume of PBS for downstream experiments or stored at −80°C.

### Determination of sEV intake

The sEVs were stained using a PKH26 red fluorescence labeling kit (UR52302, Umibio) to observe the intake of sEVs by cardiomyocytes. Briefly, the sEVs were stained with PKH26 (1:100 v/v). The labeling reaction was terminated by the addition of 0.5% bovine serum albumin after 5 min of staining. The sEVs were centrifuged at 100,000*×g* for 1 h and resuspended in 100 μl of PBS. Cardiomyocytes were incubated with 10 μg/ml PKH26-labeled sEVs for 24 h and fixed with 4% paraformaldehyde. Co-staining was performed using α-actinin (11313-2-AP, Proteintech) and DAPI. The presence of PKH26-labeled sEVs within cardiomyocytes was observed using confocal microscopy (Carl Zeiss, Germany).

### Transcriptome and enrichment analysis of sEVs

Enze Kangtai Biological Technology Co., Ltd. performed RNA sequencing using Novaseq6000. Initially, reads containing adapters, with >10% unknown bases, and low sequencing quality (<10) were eliminated. The remaining clean reads after filtering were used for bioinformatics analyses. Using bowtie2 alignment software, the clean reads were first matched to the *Mus musculus* rRNA database to filter the remaining rRNA reads. Transcriptome assembly and quantification were performed using the non-rRNA reads. The reference genome (GRCm39) and gene model annotation files were obtained from the following database: https://ftp.ensembl.org/pub/release-110/fasta/mus_musculus/dna/. The clean RNA-seq data reads were mapped to GRCm39, and HTseq-count was used to quantify gene expression levels. Genes with >10 counts across samples were retained in their raw form for subsequent analysis. Differential expression analysis was conducted using DESeq2 and edgeR, applying a *P*-value threshold of <0.05, to identify differentially expressed genes (DEGs). ClusterProfiler was used to conduct Kyoto Encyclopedia of Genes and Genomes (KEGG) pathway and Gene Ontology (GO) enrichment analyses.

### Extraction of RNA and quantitative real-time PCR (qRT-PCR)

Total RNA was extracted using TRIzol reagent (Invitrogen). Reverse transcription and qRT-PCR were performed to quantify gene expression using the HiScript Reverse Transcription Kit, ChamQ SYBR Green qPCR Kit (Vazyme), and a LightCycler 480 (Roche) system according to the manufacturer’s instructions. The 2^−△△CT^ method was used to calculate relative gene expression, and fold changes were determined using β-actin as the internal control gene. The sequences of the forward and reverse primers in the 5′–3′ direction were as follows: *Morrbid*, TCTGAGAATGAGGGGACTGG and TGTGCTGTGAAGATCCCAAG, *Serpine1*, TTCAGCCCTTGCTTGCCTC and ACACTTTTACTCCGAAGTCGGT, β-actin, ACTGCCGCATCCTCTTCCT and TCAACGTCACACTTCATGATGGA.

### Cell transfection

Transfection was performed according to a well-established protocol (34). Serpine1 short-interfering (si)RNA was introduced into the culture medium for gene knockdown at a final oligonucleotide concentration of 50 nM or a multiplicity of infection of 30.

### Statistical analysis

All experimental data were statistically analyzed using GraphPad Prism 8.0 software. Data are presented as mean ± standard error of the mean (SEM) or median (interquartile range, IQR), depending on data distribution. Normality of data distribution was first assessed using the Shapiro–Wilk test. For normally distributed data, comparisons between two groups were performed using an unpaired Student's *t* test, and comparisons among multiple groups were performed using one-way ANOVA followed by Bonferroni's post-hoc test. For non-normally distributed data, comparisons between two groups were performed using the Mann–Whitney *U* test, and comparisons among multiple groups were performed using the Kruskal–Wallis test followed by Dunn's post hoc test. A *P*-value < 0.05 was considered statistically significant, denoted as: ∗*P* < 0.05; ∗∗*P* < 0.01; ∗∗∗*P* < 0.001; ∗∗∗∗*P* < 0.0001.

## Results

### CA preserves cardiac function in mice under extreme cold stress

**Fig. 1:**
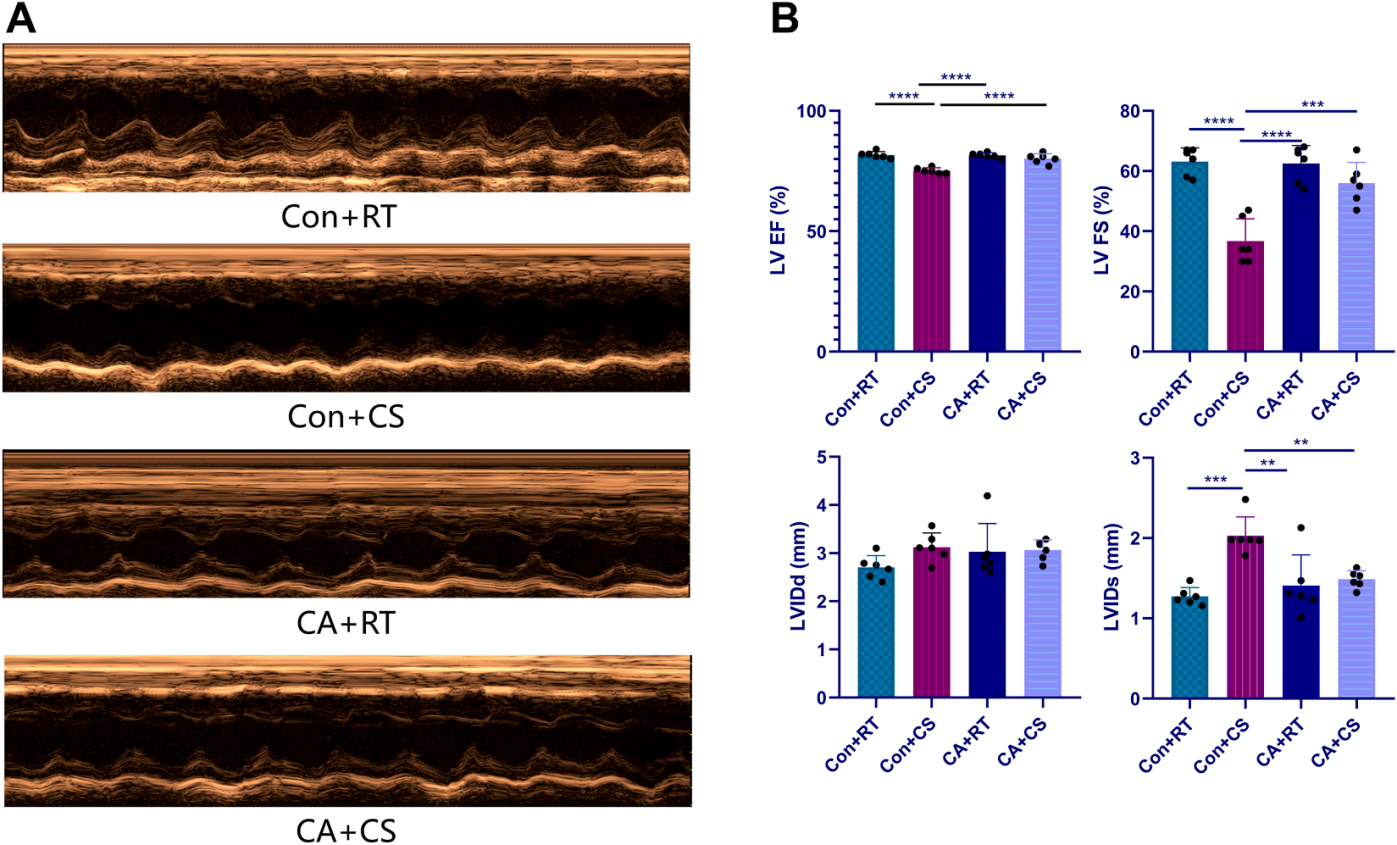
Cold acclimation alleviates cardiac dysfunction in extremely cold environments. A: Representative echocardiographic images showing the cardiac function of mice in each group. B: Quantitative analysis of LVEF, FS, LVIDd, and LVIDs (n = 6 per group). Summary data are presented as the mean ± SEM. Statistical significance was calculated using one-way ANOVA with Tukey’s multiple comparisons test; ∗*P* < 0.05, ∗∗*P* < 0.01, ∗∗∗*P* < 0.001, and ∗∗∗∗*P* < 0.0001. Abbreviations: FS, left ventricular fractional shortening; LVEF, left ventricular ejection fraction; LVIDd, left ventricular internal dimension in diastole; LVIDs, left ventricular internal dimension in systole; CA, cold acclimation; RT, room temperature; CS, cold stress.

Mice were divided into four groups (n = 6/group): Con + RT (untrained control, 25°C), Con + CS (untrained control, extreme cold stress at −25°C), CA + RT (cold acclimation, 25°C), and CA + CS (cold acclimation, −25°C). Echocardiography revealed that extreme cold exposure (Con + CS) induced myocardial contractile dysfunction, characterized by diminished left ventricular (LV) wall motion and systolic chamber dilation, compared to the Con + RT group (Fig. 1A). Quantitatively, Con + CS mice exhibited significantly decreased LVEF and LVFS (both *P* < 0.0001) along with increased LVIDs (*P* < 0.001) (Fig. 1B). Conversely, prior cold acclimation (CA + CS) effectively preserved cardiac function, as evidenced by restored LVEF (*P* < 0.0001) and LVFS (*P* < 0.001) and reduced LVIDs (*P* < 0.01) compared to the Con + CS group. The changes in LVIDd followed similar but milder trends.

**Fig. 2:**
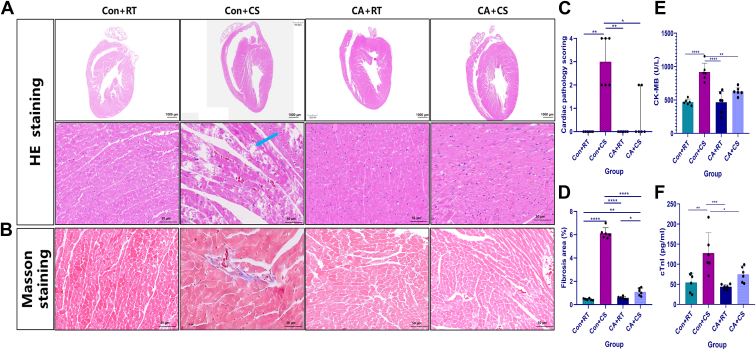
Cold acclimation alleviates myocardial injury in extremely cold environments. A: Representative images of H&E staining (scale bar = 50 μm). B: Representative images of Masson’s staining (scale bar = 50 μm). C: Cardiac pathology scoring analysis of H&E staining (n = 6 per group); statistical significance was calculated using the Kruskal–Wallis test followed by Dunn’s post-hoc test. D: Quantitative analysis of Masson’s staining (n = 6 per group). Statistical significance was calculated using one-way ANOVA with Tukey’s multiple comparison test. E, F: Serum CK-MB and cTnI levels were measured in each group (n = 6 per group), and statistical significance was calculated using one-way ANOVA with Tukey’s multiple comparisons test. Summary data are presented as the mean ± SEM or median (IQR).∗*P* < 0.05, ∗∗*P* < 0.01, ∗∗∗*P* < 0.001, ∗∗∗∗*P* < 0.0001. Abbreviations: CK-MB, creatine kinase-MB; cTnI, cardiac troponin I; H&E, hematoxylin and eosin.

Histological analysis further confirmed the protective effects of CA (Fig. 2). H&E staining (Fig. 2A) showed that Con + CS hearts suffered acute focal myocardial injury with disorganized and fragmented myofibers (blue arrows), leading to a significantly elevated cardiac pathology score (*P* < 0.01 vs. Con + RT) (Fig. 2C). Masson’s trichrome staining revealed increased myocardial collagen deposition and fibrotic area in the Con + CS group (Fig. 2B, D) (*P* < 0.0001). Importantly, CA attenuated this structural damage, reducing both the pathology score (*P* < 0.05) and the fibrotic area (*P* < 0.0001) in CA + CS mice. Consistently, serum levels of myocardial injury markers, CK-MB and cTnI (Fig. 2E, F), were elevated following extreme cold exposure (*P* < 0.0001 and *P* < 0.01 vs. Con + RT, respectively). However, cold acclimation did not induce this release, with CA + CS mice showing lower CK-MB (*P* < 0.01) and cTnI (*P* < 0.05) levels than the Con + CS group.

### BAT is indispensable for CA-induced cardioprotection

Given the central role of BAT in CA, we hypothesized that it mediates cardioprotection. Consistent with its function as the primary thermogenic organ, BAT mass was significantly increased in CA mice compared to that in controls (Supplemental Fig. S1A–C). This expansion was accompanied by functional activation, as evidenced by the marked upregulation of UCP1 (Supplemental Fig. S1D). Morphologically, CA induced robust remodeling characterized by a reduction in the size of multilocular lipid droplets and a diffuse, high-intensity UCP1 immunofluorescent signal (Supplemental Fig. S1E, F), confirming a shift toward a hypermetabolic phenotype. To determine the necessity of BAT for CA-mediated cardioprotection, we established an iBAT surgical ablation model and assessed cardiac function using echocardiography, serum biomarkers of myocardial injury, and histopathological evaluation. Notably, the cold stress temperature was adjusted to −15°C in these experiments, as the original −25°C protocol resulted in premature mortality in experimental animals (as shown in Supplemental Fig S2, exposure to −15°C had no statistically significant effect on myocardial injury differences between the CA group and Con group mice).

**Fig. 3:**
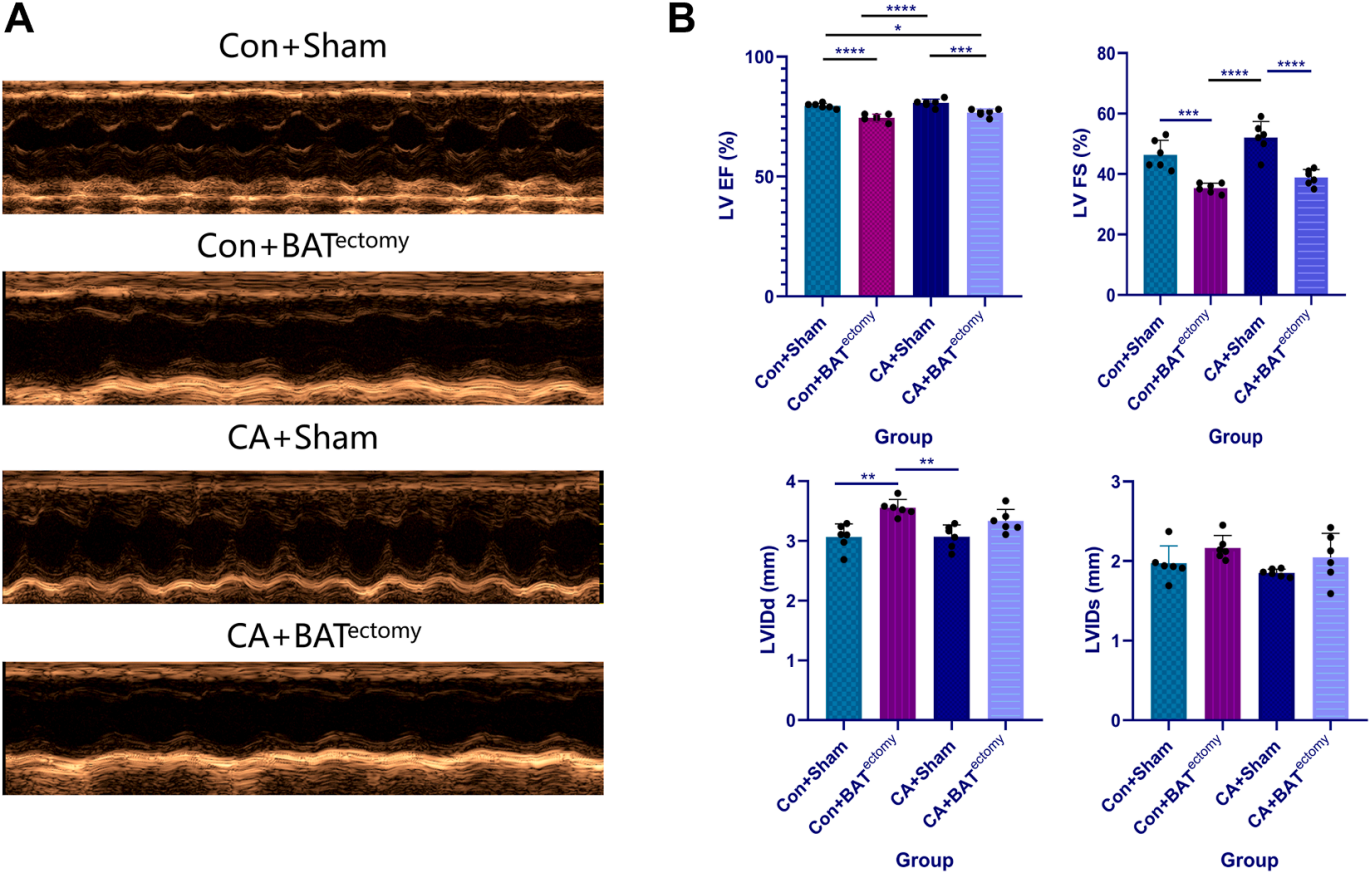
The removal of iBAT diminished the protective effect of cold acclimation on myocardial function in mice exposed to extreme cold conditions. A: Representative echocardiographic images showing the cardiac function of mice in each group. B: Quantitative analysis of LVEF, FS, LVIDd, and LVIDs (n = 6 per group). Summary data are presented as the mean ± SEM. Statistical significance was calculated using one-way ANOVA with Tukey’s multiple comparisons test; ∗*P* < 0.05, ∗∗*P* < 0.01, ∗∗∗*P* < 0.001, and ∗∗∗∗*P* < 0.0001. Abbreviations: FS, left ventricular fractional shortening; LVEF, left ventricular ejection fraction; LVIDd, left ventricular internal dimension in diastole; LVIDs, left ventricular internal dimension in systole; CA, cold acclimation; RT, room temperature; CS, cold stress; BAT, brown adipose tissue.

Echocardiography (Fig. 3A) revealed that, although cold-acclimated sham mice (CA + Sham) maintained ventricular wall motion, iBAT^ectomy^ abolished this protective effect, resulting in systolic chamber dilation in the CA + iBAT^ectomy^ group. Quantitatively (Fig. 3B), preserved LVEF and LVFS in CA + Sham mice were reduced following iBAT^ectomy^ (*P* < 0.0001 vs. CA + Sham). Furthermore, the CA-mediated prevention of ventricular dilation was reversed, as evidenced by a significant increase in LVIDs in the CA + iBAT^ectomy^ group (*P* < 0.01 vs. CA + Sham).

**Fig. 4:**
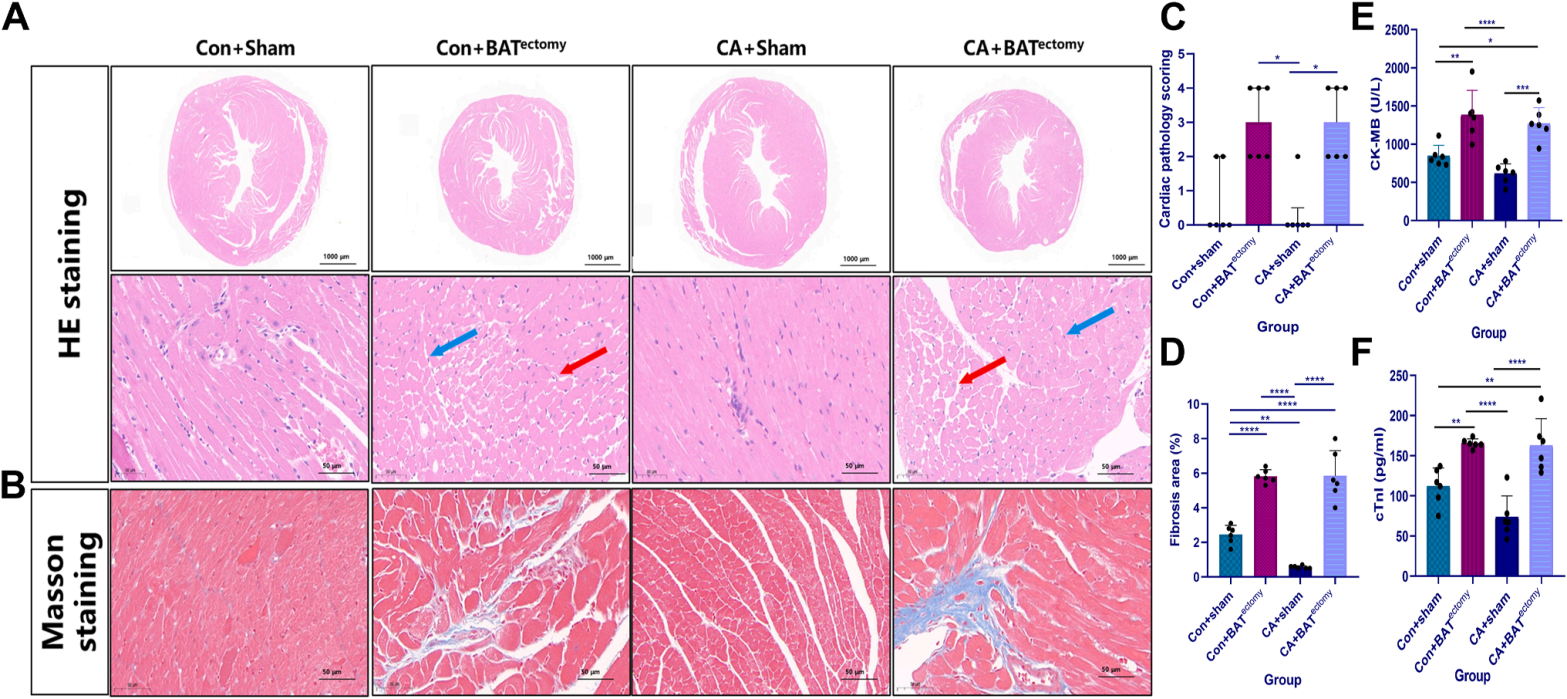
Cold-acclimated mice developed myocardial injury under extreme cold conditions after iBAT removal. A: Representative images of H&E staining (scale bar = 50 μm). B: Representative images of Masson’s staining (scale bar = 50 μm). C: Cardiac pathology scoring analysis of H&E staining (n = 6 per group), statistical significance was calculated using Kruskal–Wallis test followed by Dunn’s post-hoc test. D: Quantitative analysis of Masson’s staining (n = 6 per group), statistical significance was calculated using one-way ANOVA with Tukey’s multiple comparisons test. E, F: Serum CK-MB and cTnI levels were measured in each group (n = 6 per group), statistical significance was calculated using one-way ANOVA with Tukey’s multiple comparisons test. Summary data are presented as the mean ± SEM or median (IQR).∗*P* < 0.05, ∗∗*P* < 0.01, ∗∗∗*P* < 0.001, ∗∗∗∗*P* < 0.0001. Abbreviations: CK-MB, creatine kinase-MB; cTnI, cardiac troponin I; H&E, hematoxylin and eosin.

Histopathological and biomarker analyses further confirmed the indispensability of iBAT (Fig. 4). H&E staining (Fig. 4A) showed cold-induced myofiber fragmentation and degeneration in control mice. While CA protected the myocardial tissue, iBAT removal re-exposed the CA mice to structural damage, leading to a significantly elevated cardiac pathology score (*P* < 0.05 vs. CA + Sham) (Fig. 4C). Masson’s staining (Fig. 4B, D) revealed that the minimal collagen deposition seen in CA + Sham hearts was replaced by marked myocardial fibrosis following iBAT removal (*P* < 0.0001 vs. CA + Sham). Consistent with this finding, the low serum levels of myocardial injury markers (CK-MB and cTnI) maintained by CA were elevated in the CA + iBAT^ectomy^ group (*P* < 0.001 and *P* < 0.0001 vs. CA + Sham, respectively) (Fig. 4E, F), indicating cardiomyocyte injury.

### Circulating EVs contribute to the cardioprotective efficacy of CA

**Fig. 5:**
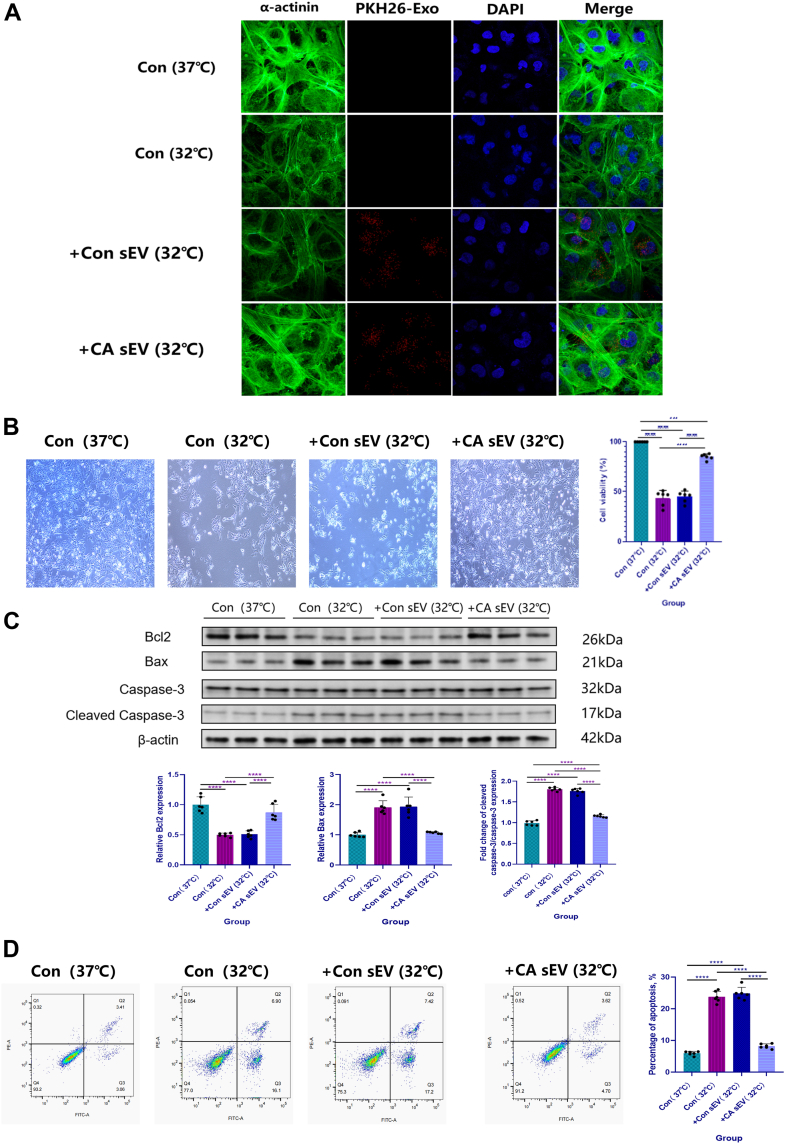
sEVs from cold-acclimated mice protect HL-1 cardiomyocytes from hypothermia-induced damage. A: PKH26 labeling for the detection of cardiomyocyte internalization of exosomes derived from CA mice (scale bar = 15 μm); B: Morphological image (10×) and CCK-8 cell viability assays of different HL-1 cell groups; C: Cardiac protein expression of Bcl2, Bax, casepase-3 and cleaved casepase-3 was measured by Western blot (n = 6 per group); D: Flow cytometry and quantitative analysis of Annexin V and PI in different HL-1 cell groups (n = 6 per group). Summary data are presented as the mean ± SEM. Statistical significance was calculated using one-way ANOVA with Tukey’s multiple comparisons test; ∗*P* < 0.05, ∗∗*P* < 0.01, and ∗∗∗∗*P* < 0.0001. Abbreviations: sEVs, Small extracellular vesicles.

Having established that iBAT is indispensable for CA-induced cardioprotection, we hypothesized that this remote effect is mediated by circulating EVs. To investigate this phenomenon, we isolated serum EVs from CA and Con mice and characterized them using western blotting and nanoflow cytometry. As illustrated in Supplemental Fig. S3A, B, EVs expressing canonical markers (Alix, CD9, and HSP70) were detected in both groups, with a predominant diameter ranging from 60 to100 nm. Next, we evaluated the functional capacity of these EVs using an in vitro cold stress model. HL-1 cardiomyocytes were treated with sEVs (20 μg/ml) isolated from either CA or Con mice. Fluorescence imaging confirmed the efficient internalization of the EVs by HL-1 cells (Fig. 5A). Importantly, while Con-derived EVs failed to confer protection, treatment with CA-derived EVs significantly attenuated cold-induced injury as evidenced by preserved cell viability and reduced apoptosis (Fig. 5B–D). These findings indicate that CA qualitatively alters the bioactive cargo of circulating EVs to confer cardioprotective effects.

### BAT-derived lncRNA Morrbid is essential for CA-mediated cardioprotection

**Fig. 6:**
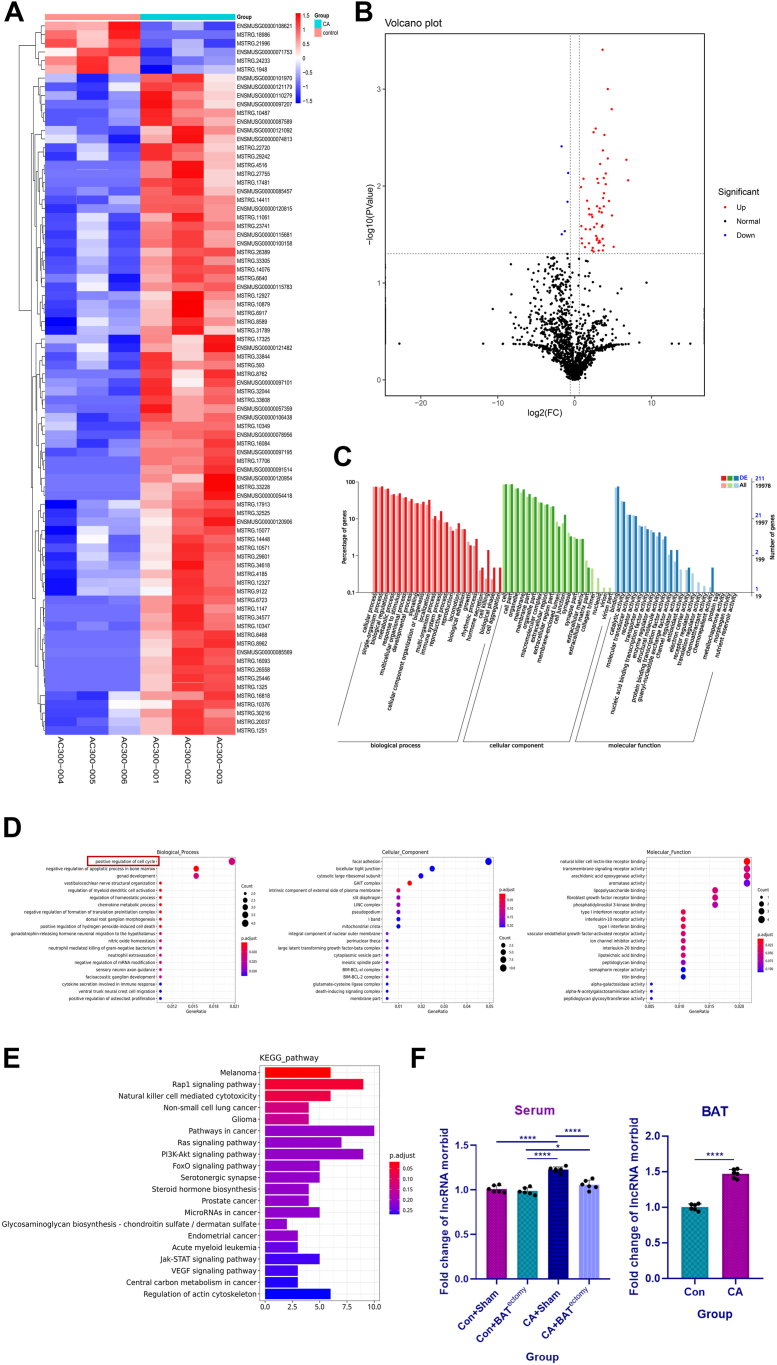
LncRNA *Morrbid* was enriched in iBAT sEVs and serum sEVs. A: Heat maps of the hierarchical clustering analysis of overall DE-lncRNAs, Cut-off values: *P* < 0.05, fold change>1.5; B: Volcano plot of DE-lncRNAs in the two groups (n = 3 per group). C: GO functional annotation of the target genes of DE-lncRNAs. D: Top 10 GO items for biological processes, molecular functions, and cellular components. E: KEGG pathway enrichment of the target genes of DE-lncRNAs. F: RT-PCR analysis of candidate lncRNAs in iBAT sEVs and serum sEVs of CA or control mice. (n = 6). An unpaired, 2-tailed Student t *t* test was used to analyze the data; ∗*P* < 0.05. Abbreviations: DE-lncRNAs, differentially expressed lncRNAs; RT-PCR, real-time polymerase chain reaction; sEVs, small extracellular vesicles; iBAT, interscapular brown adipose tissue.

Considering that EVs carry diverse molecular cargoes, we hypothesized that specific RNA species might drive the observed protective effects. To identify these potential candidates, we performed transcriptomic profiling of EVs from both groups. Comparative analysis identified 82 differentially expressed lncRNAs, 76 of which were significantly upregulated in the CA-derived EVs (Fig. 6A–B). Functional enrichment analyses (GO and KEGG) revealed that the target genes of these lncRNAs were predominantly associated with “positive regulation of the cell cycle” (Fig. 6C–E). Among the candidates linked to this functional term, combined with previous literature indicating its role in cardiac stress responses, lncRNA *Morrbid* was selected as the key candidate of interest. Quantitative PCR validation confirmed that Morrbid levels were significantly elevated in both BAT- and serum-derived EVs from CA mice (Fig. 6F), suggesting its potential role as a cardioprotective effector.

To determine whether lncRNA *Morrbid* is necessary for this protection, we performed a loss-of-function study by injecting AAV9-sh*Morrbid* or AAV9-negative control (AAV9-NC) into the iBAT of CA mice. This approach successfully achieved and isolated sEVs for the subsequent treatment of HL-1 cardiomyocytes. To confirm that AAV administration did not impair the thermogenic function of iBAT, we evaluated iBAT tissues from all groups using immunofluorescence, H&E staining, and western blotting (Supplemental Fig. S4).

**Fig. 7:**
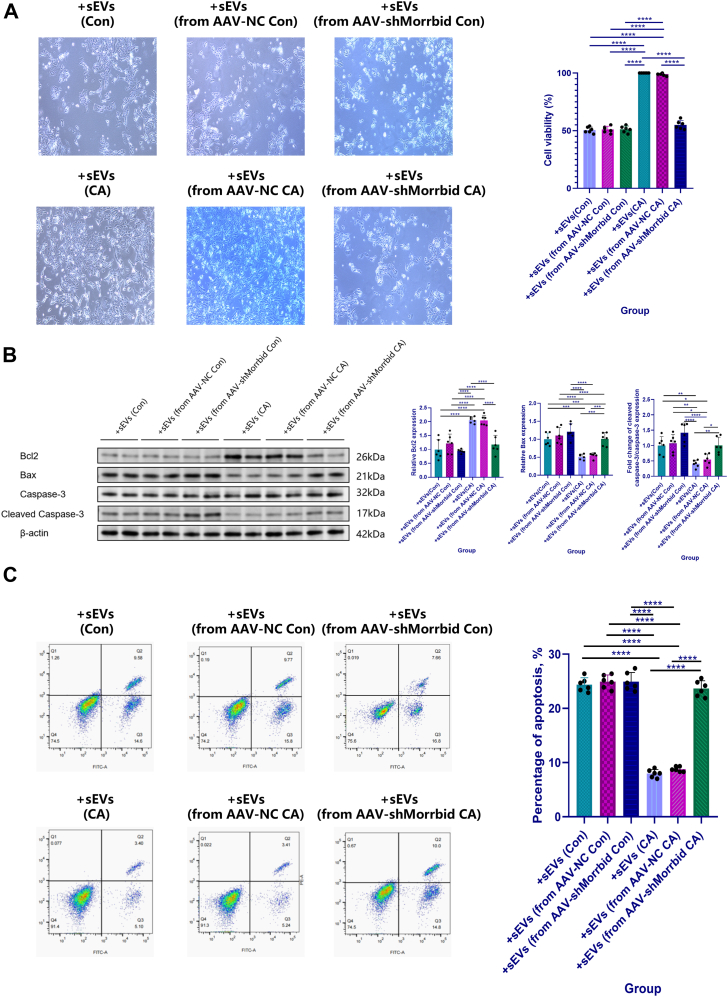
The lncRNA *Morrbid* is the key molecule mediating the protective effects of CA sEVs on cardiomyocyte survival. A: Morphological image (10×) and CCK-8 cell viability assays of different HL-1 cell groups. B: Cardiac protein expression of Bcl2, Bax, casepase-3 and cleaved casepase-3 was measured by Western blot (n = 6 per group). C: Flow cytometry and quantitative analysis of Annexin V and PI in different HL-1 cell groups (n = 6 per group). Summary data are presented as the mean ± SEM. Statistical significance was calculated using one-way ANOVA with Tukey’s multiple comparisons test; ∗*P* < 0.05, ∗∗∗*P* < 0.001.

The functional impact of *Morrbid*-enriched EVs was assessed using CCK-8 cell viability assays, western blotting analysis of apoptosis-related proteins, and flow cytometry-based detection of apoptosis. As shown in Supplemental Fig. S5, AAV9-sh*Morrbid* was successfully delivered into iBAT, leading to decreased *Morrbid* expression in sEVs. Under cold stress, HL-1 cells treated with EVs from AAV9-sh*Morrbid*-injected CA mice exhibited significantly reduced viability (Fig. 7A). Western blotting analysis further revealed the downregulation of the anti-apoptotic protein Bcl-2 and upregulation of the pro-apoptotic proteins Bax and Cleaved caspase-3 in this group (Fig. 7B). Consistent with these findings, flow cytometry confirmed a marked increase in the apoptosis rate of HL-1 cells treated with EVs from *Morrbid*-knockdown CA mice (Fig. 7C).

### Serpine1 serves as a critical downstream effector of Morrbid

**Fig. 8:**
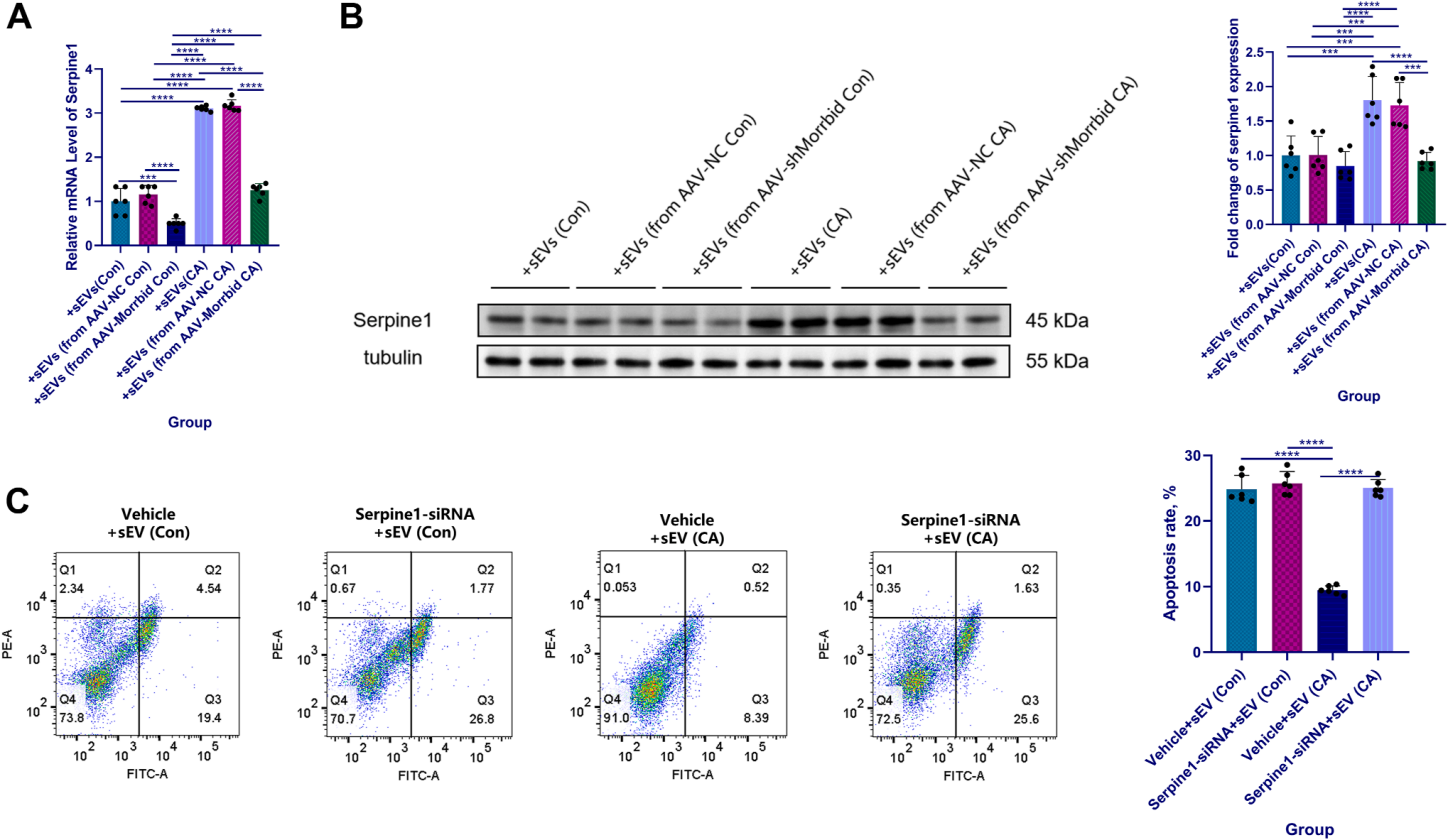
The lncRNA Morrbid potentially promotes cardiomyocyte survival by targeting Serpine1. A: Expression of Serpine1 in HL-1 cells was measured by RT-qPCR (n = 6 per group). B: Protein expression of Serpine1 in HL-1 cells was detected by Western blot (n = 6 per group). C: Flow cytometry and quantitative analysis of Annexin V and PI in different HL-1 cell groups (n = 6 per group). Summary data are presented as the mean ± SEM. Statistical significance was calculated using one-way ANOVA with Tukey’s multiple comparisons test; ∗*P* < 0.05, ∗∗∗*P* < 0.001.

**Fig. 9:**
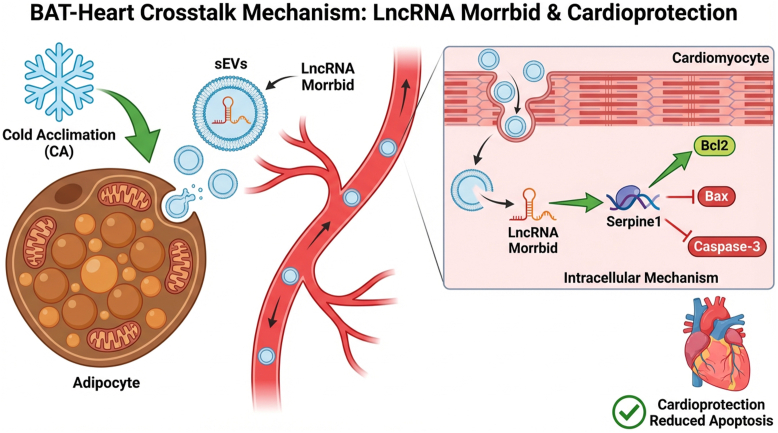
Schematic illustration of the working model of this study.

Mechanistically, previous studies suggest that *Morrbid* modulates the apoptosis of cardiomyocytes by regulating Serpine1 (35). To investigate this downstream pathway, we first measured the protein expression level of Serpine1 in mouse cardiomyocytes. Treatment with sEVs derived from *Morrbid*-knockdown CA mice resulted in a significant downregulation of Serpine1 at both mRNA and protein levels (Fig. 8A–B). To verify the functional necessity of Serpine1 for cardiomyocyte survival, siRNA-mediated silencing was performed. Following effective knockdown (Supplemental Fig. S6), Serpine1-depleted cardiomyocytes exhibited a significant increase in the apoptosis rate (Fig. 8C). These findings suggest that Serpine1 is a potential downstream effector contributing to Morrbid-mediated cardioprotection. The proposed BAT-Heart axis and molecular mechanism are schematically summarized in Figure 9.

## Discussion

In this study, we provide the first evidence that CA significantly enhances cardiac resistance to extreme environmental cold stress in mice. We demonstrated that this protective effect is closely associated with sEVs, with iBAT serving as an important cellular source for delivering lncRNA Morrbid. This suggests that its action may partially occur via the regulation of Serpine1 expression in cardiomyocytes. These findings offer new insights into the novel inter-organ communication axis between BAT and the heart and identify a potential molecular target for mitigating environmental cold stress-induced myocardial injury.

Previous studies have established that CA improves cold resistance and modulates energy metabolism and the oxidative stress response. Recent research has revealed that CA elevates the circulating levels of omega-6 and omega-3 oxylipins in humans, which correlates with a healthier cardiometabolic profile (36). Concurrently, studies in mice have shown that cold exposure activates cardiac β_2_/β_3_-adrenergic receptors, mitigating myocardial IRI and improving cardiac function (11, 12). However, most existing studies view CA primarily as a preconditioning strategy for clinical ischemic events. A critical gap remains regarding whether CA can directly shield the heart from injury caused by environmental hypothermia itself. Building on this foundation, our study addresses this gap by demonstrating that extreme cold exposure can directly induce cardiac injury and that CA serves as an effective strategy to attenuate such damage.

In 2015, Thoonen *et al.* first reported, via adipose transplantation experiments, that scapular BAT participates in regulating cardiac remodeling and that BAT from UCP1-knockout mice failed to confer cardioprotection (37). In 2018, Pingjin *et al.* demonstrated that in hypertensive cardiac remodeling, the activation of adenosine A_2_A receptors on brown adipocytes stimulates FGF21 secretion via the AMPK/PGC1α pathway, thereby alleviating myocardial remodeling (21). The same team reported in 2022 that during cardiac hypertrophy and fibrosis, the activation of β_3_-adrenergic receptors on brown adipocytes suppresses the release of exosomes containing inducible nitric oxide synthase via the ERK1/2 pathway, mitigating pathological myocardial changes (38). Recently, Marielle *et al.* showed that BAT activation during myocardial IRI leads to the secretion of bone morphogenetic protein 3, which attenuates IRI via the Smad1/5/8 signaling pathway (39). Collectively, these pioneering studies have established that BAT communicates with the heart primarily through protein-based “batokines.” However, whether non-coding RNAs, particularly lncRNAs, can serve as functional cargo in this inter-organ dialogue remains unexplored. Zhao *et al.* reported that exercise-induced BAT releases EVs enriched with miR-125b-5p, miR-128-3p, and miR-30d-5p, contributing to protection against myocardial IRI (40). Extending beyond this miRNA-centric view, we performed a transcriptomic analysis of sEVs from CA mice and identified 82 DEGs. In contrast to previous approaches that prioritized miRNAs, this study specifically aimed to identify functionally relevant lncRNAs. Although miRNAs are more commonly studied and are often more abundant in EVs, lncRNAs are increasingly recognized for their multilayered roles in gene regulatory networks, including chromatin remodeling, transcriptional regulation, and protein scaffolding, underscoring the relevance of our research focus.

Transcriptomic analysis revealed a significant upregulation of lncRNA *Morrbid* in sEVs from CA mice. Previous studies have shown that stress-induced upregulation of *Morrbid* in cardiac tissue alleviates myocardial infarction and improves cardiac function (35). Subsequent qPCR validation confirmed higher *Morrbid* expression levels in both the serum and BAT of CA mice, indicating that CA induces *Morrbid* expression in BAT and promotes its secretion into the circulation via EVs. To the best of our knowledge, this is the first study to identify iBAT as an important secretory source of lncRNA *Morrbid* and to establish it as a novel long-distance signaling molecule in inter-organ communication during CA. This finding expands the concept of the BAT secretome from proteins and miRNAs to include lncRNAs, adding a new layer of complexity to our understanding of the “BAT-Heart” axis. Consistent with previous studies (35), this study identified Serpine1 as a potential target for *Morrbid* binding and anti-apoptotic effects. Future development of drugs targeting this site may enhance myocardial cell survival under hypothermic conditions.

This study has several limitations. First, due to the extremely high mortality of iBAT-ablated mice under severe cold stress (−25°C), we employed moderate cold stress (−15°C) in our surgical models. Importantly, control mice at −15°C showed no significant differences in cardiac damage between the CA and Con groups, confirming that injury severity is temperature-dependent. Second, as our surgical approach targeted only iBAT, our conclusions reflect iBAT function rather than total BAT. Third, although we demonstrated in vitro cardiomyocyte uptake of BAT-derived EVs, direct in vivo evidence for their specific cardiac targeting is technically challenging and warrants future biodistribution analyses. Fourth, although SEC provides superior EV purity compared to precipitation methods, it cannot completely eliminate co-isolated lipoproteins of overlapping size (e.g., VLDL and chylomicrons), and negative markers (e.g., ApoA1, ApoB) were not assessed. Therefore, the potential biological contribution of co-isolated lipoproteins to the observed effects cannot be entirely excluded, and the isolated particles should be considered sEV-enriched fractions. Fifth, core body temperature was not monitored during cold exposure, as repeated handling for measurement would introduce confounding stress; thus, thermoregulatory kinetics between experimental conditions were not characterized. Finally, the detailed molecular mechanism by which Serpine1 regulates anti-apoptotic effects requires further investigation.

## Conclusion

In summary, this study revealed a novel mechanism by which BAT communicates with the heart during CA. We propose that iBAT serves as a critical cellular source for the secretion of sEV-enriched fractions enriched with lncRNA *Morrbid*, which may subsequently upregulate Serpine1 in cardiomyocytes, potentially alleviating apoptosis and contributing to the protection of cardiac function under cold stress.

## Ethics Approval and Consent to Participate

The Research Ethics Committee of Chinese People’s Liberation Army General Hospital approved this study.

## Data availability

The datasets used and/or analyzed during the current study are available from the corresponding author on reasonable request.

## Supplemental data

This article contains supplemental data.

## Conflict of interest

The authors declare that they have no conflicts of interest with the contents of this article.

## References

[1] Chen J., Gao Y., Jiang Y., Li H., Lv M., Duan W. (2022). Low ambient temperature and temperature drop between neighbouring days and acute aortic dissection: a case-crossover study. Eur. Heart J..

[2] Huang J., He Q., Jiang Y., Wong J.M.J., Li J., Liu J. (2025). Low ambient temperature and incident myocardial infarction with or without obstructive coronary arteries: a Chinese nationwide study. Eur. Heart J..

[3] Kazi D.S., Katznelson E., Liu C.L., Al-Roub N.M., Chaudhary R.S., Young D.E. (2024). Climate change and cardiovascular health: a systematic review. JAMA Cardiol..

[4] Cong P., Liu Y., Liu N., Zhang Y., Tong C., Shi L. (2018). Cold exposure induced oxidative stress and apoptosis in the myocardium by inhibiting the Nrf2-Keap1 signaling pathway. BMC Cardiovasc. Disord..

[5] Zhang J., You S., Yu L., Zhang Y., Li Z., Zhao N. (2024). The hysteresis damage of cold exposure on tissue and transcript levels in mice. J. Therm. Biol..

[6] Zhou M.G., Wang L.J., Liu T., Zhang Y.H., Lin H.L., Luo Y. (2014). Health impact of the 2008 cold spell on mortality in subtropical China: the climate and health impact national assessment study (CHINAs). Environ. Health.

[7] Wang L., Liu T., Hu M., Zeng W., Zhang Y., Rutherford S. (2016). The impact of cold spells on mortality and effect modification by cold spell characteristics. Sci. Rep..

[8] Guan X., Gao Z., Huang J., Cao C., Zhu K., Wang J. (2022). Speeding extreme cold events under global warming. Environ. Res. Lett..

[9] Johnson N.C., Xie S.P., Kosaka Y., Li X. (2018). Increasing occurrence of cold and warm extremes during the recent global warming slowdown. Nat. Commun..

[10] Gordon K., Blondin D.P., Friesen B.J., Tingelstad H.C., Kenny G.P., Haman F. (2019). Seven days of cold acclimation substantially reduces shivering intensity and increases nonshivering thermogenesis in adult humans. J. Appl. Physiol. (1985).

[11] Tibenska V., Benesova A., Vebr P., Liptakova A., Hejnová L., Elsnicová B. (2020). Gradual cold acclimation induces cardioprotection without affecting β-adrenergic receptor-mediated adenylyl cyclase signaling. J. Appl. Physiol. (1985).

[12] Tibenska V., Marvanova A., Elsnicova B., Hejnova L., Vebr P., Novotný J. (2021). The cardioprotective effect persisting during recovery from cold acclimation is mediated by the β(2)-adrenoceptor pathway and Akt activation. J. Appl. Physiol. (1985).

[13] Voronkov N.S., Popov S.V., Naryzhnaya N.V., Prasad N.R., Petrov I.M., Kolpakov V.V. (2024). Effect of cold adaptation on the State of cardiovascular System and cardiac tolerance to Ischemia/Reperfusion Injury. Iran Biomed. J..

[14] Tsibulnikov S.Y., Maslov L.N., Naryzhnaya N.V., Ivanov V.V., Bushov Y.V., Voronkov N.S. (2019). Impact of cold adaptation on cardiac tolerance to ischemia/reperfusion and glucocorticoid, thyroid hormone levels. Gen. Physiol. Biophys..

[15] Marvanova A., Kasik P., Elsnicova B., Tibenska V., Galatik F., Hornikova D. (2023). Continuous short-term acclimation to moderate cold elicits cardioprotection in rats, and alters β-adrenergic signaling and immune status. Sci. Rep..

[16] Lidell M.E., Betz M.J., Enerbäck S. (2014). Brown adipose tissue and its therapeutic potential. J. Intern. Med..

[17] Scheele C., Wolfrum C. (2020). Brown adipose crosstalk in tissue plasticity and human metabolism. Endocr. Rev..

[18] Gavaldà-Navarro A., Villarroya J., Cereijo R., Giralt M., Villarroya F. (2022). The endocrine role of brown adipose tissue: an update on actors and actions. Rev. Endocr. Metab. Disord..

[19] Ding Y., Su J., Shan B., Fu X., Zheng G., Wang J. (2024). Brown adipose tissue-derived FGF21 mediates the cardioprotection of dexmedetomidine in myocardial ischemia/reperfusion injury. Sci. Rep..

[20] Feng L., Cui J., Chen W., Zhu L., Li P., Zhou H. (2025). Nrg4 secreted by brown adipose tissue suppresses ferroptosis of sepsis-induced liver injury. Inflammation.

[21] Ruan C.C., Kong L.R., Chen X.H., Ma Y., Pan X.X., Zhang Z.B. (2018). A(2A) receptor activation attenuates hypertensive cardiac remodeling via promoting brown adipose tissue-derived FGF21. Cell Metab..

[22] Chen Y., Pfeifer A. (2017). Brown fat-derived exosomes: small vesicles with big impact. Cell Metab..

[23] Thomou T., Mori M.A., Dreyfuss J.M., Konishi M., Sakaguchi M., Wolfrum C. (2017). Adipose-derived circulating miRNAs regulate gene expression in other tissues. Nature.

[24] Sun Z., Li J., Zheng Y., Zhang J., Bai W., Deng X. (2025). Small extracellular vesicles derived from mesenchymal stromal cells loaded with β-nicotinamide mononucleotide activate NAD+/SIRT3 signaling pathway-mediated mitochondrial autophagy to delay skin aging. Stem Cell Res. Ther..

[25] Jin C., Wu P., Wu W., Chen W., Liu W., Zhu Y. (2025). Therapeutic role of hucMSC-sEV-enriched miR-13896 in cisplatin-induced acute kidney injury through M2 macrophage polarization. Cell Biol. Toxicol.

[26] Semeradtova A., Liegertova M., Herma R., Capkova M., Brignole C., Del Zotto G. (2025). Extracellular vesicles in cancer's communication: messages we can read and how to answer. Mol. Cancer.

[27] Kuang L., Wu L., Li Y. (2025). Extracellular vesicles in tumor immunity: mechanisms and novel insights. Mol. Cancer.

[28] Rohm T.V., Cunha E.R.K., Olefsky J.M. (2025). Metabolic Messengers: small extracellular vesicles. Nat. Metab..

[29] Liu Y., Xue N., Zhang B., Lv H., Li S. (2022). Cold stress induced liver injury of mice through activated NLRP3/Caspase-1/GSDMD pyroptosis signaling pathway. Biomolecules.

[30] Rona G., Chappel C.I., Balazs T., Gaudry R. (1959). An infarct-like myocardial lesion and other toxic manifestations produced by isoproterenol in the rat. AMA Arch. Pathol..

[31] Cui J., Chen Y., Yang Q., Zhao P., Yang M., Wang (2024). X.,Protosappanin A protects DOX-Induced myocardial injury and cardiac dysfunction by targeting ACSL4/FTH1 axis-dependent ferroptosis. Adv. Sci. (Weinh).

[32] Hu Y., Yu J., Cui X., Zhang Z., Li Q., Guo W. (2021). Combination usage of AdipoCount and image-pro Plus/ImageJ software for quantification of adipocyte sizes. Front Endocrinol. (Lausanne).

[33] Tai P., Chen X., Jia G., Chen G., Gong L., Cheng Y. (2023). WGX50 mitigates doxorubicin-induced cardiotoxicity through inhibition of mitochondrial ROS and ferroptosis. J. Transl Med..

[34] Cheng Y., Ji R., Yue J., Yang J., Liu X., Chen H. (2007). MicroRNAs are aberrantly expressed in hypertrophic heart: do they play a role in cardiac hypertrophy?. Am. J. Pathol..

[35] Yu Y., Yang H., Li Q., Ding N., Gao J., Qiao G. (2023). Stress-enhanced cardiac lncRNA Morrbid protects hearts from acute myocardial infarction. JCI Insight.

[36] Jurado-Fasoli L., Sanchez-Delgado G., Di X., Yang W., Kohler I., Villarroya F. (2024). Cold-induced changes in plasma signaling lipids are associated with a healthier cardiometabolic profile independently of brown adipose tissue. Cell Rep. Med..

[37] Thoonen R., Ernande L., Cheng J., Nagasaka Y., Yao V., Miranda-Bezerra A. (2015). Functional brown adipose tissue limits cardiomyocyte injury and adverse remodeling in catecholamine-induced cardiomyopathy. J. Mol. Cell Cardiol..

[38] Lin J.R., Ding L.L., Xu L., Huang J., Zhang Z.B., Chen X.H. (2022). Brown adipocyte ADRB3 mediates cardioprotection via suppressing exosomal iNOS. Circ. Res..

[39] Martí-Pàmies Í., Thoonen R., Morley M., Graves L., Tamez J., Caplan A. (2023). Brown adipose tissue and BMP3b decrease injury in Cardiac ischemia-reperfusion. Circ. Res..

[40] Zhao H., Chen X., Hu G., Li C., Guo L., Zhang L. (2022). Small extracellular vesicles from brown adipose tissue mediate exercise cardioprotection. Circ. Res..

