# Supplementary material for: Brown adipose tissue-derived sEV-lncRNA Morrbid contributes to cold acclimation-induced cardioprotection against extreme cold
Source: J Lipid Res. 2026 Jun 19;67(8):101082. doi: 10.1016/j.jlr.2026.101082 (PMC13473955; doi:10.1016/j.jlr.2026.101082)
Supplement: Supplementary file 1 [file mmc1.docx]

## Supplemental Material

S1Figure. iBAT in CA mice was acticvated. A-C) body weights, iBAT weights, iBAT weight to body weight ratios (n = 7 per group); D) Protein expression of UCP1 was measured by western blot (n = 6 per group); E) Representative images and quantitative analysis of mean lipid droplet area in H&E staining (scale bar = 50μm; n = 3 per group); F) Representative images and quantitative analysis of immunohistochemical staining of UCP1 in iBAT sections (scale bar = 50μm; n = 3 per group). Summary data are presented as the mean±SEM. Statistical signiﬁcance was calculated using one-way ANOVA with Tukey’s multiple comparisons test; **P* < 0.05, ****P* < 0.001.

S2Figure. Intermittent cold exposure (-15℃, 2 h/day for 5 days) does not induce distinct structural myocardium damage in CA and Con groups. A) Representative images of H&E staining (scale bar = 50μm). B) Representative images of Masson’s staining (scale bar = 50μm). C) Cardiac pathology scoring analysis of H&E staining (n = 6 per group). D) Quantitative analysis of Masson’s staining (n = 6 per group). Statistical signiﬁcance was calculated using Kruskal-Wallis test followed by Dunn’s post-hoc test; ****P* < 0.001. Abbreviations: H&E, hematoxylin and eosin.

S3Figure. Characteristics of extracellular vesicles in brown adipose tissue of cold acclimation mice. A) Representative western blot detection of exosome markers expression; B) Representative nanoflow cytometric detection of exosome particle size distribution.

S4Figure. AAV injection does not impair brown adipose tissue thermogenic function and morphological remodeling in cold-acclimated mice. A) Representative Western blot images and corresponding quantitative analysis of UCP1 protein expression in the iBAT across the indicated groups (n = 6 per group). B) Representative H&E staining images to assess lipid droplet morphology (top panels), combined with UCP1 (red) and nucleal DAPI (blue) immunofluorescence staining (bottom panels) of iBAT sections (scale bars = 50 μm). The bar charts on the right present the quantitative analysis of adipocyte area (upper right) and UCP1 fluorescence intensity (lower right) (n = 6 per group). Statistical significance was calculated using one-way ANOVA followed by Tukey’s post-hoc test, ****P* < 0.001, *****P* < 0.0001. Abbreviations: AAV, adeno-associated virus; BAT, brown adipose tissue; CA, cold acclimation; Con, control; H&E, hematoxylin and eosin; UCP1, uncoupling protein 1; DAPI, 4’,6-diamidino-2-phenylindole.

S5Figure. LncRNA Morrbid was knocked down in CA mice by RT-PCR (n = 6 per group). Summary data are presented as the mean±SEM. Statistical signiﬁcance was calculated using one-way ANOVA with Tukey’s multiple comparisons test; **p* < 0.05.

S6Figure. Serpine1 was knocked down in HL-1 cells. A) Serpine1 was knocked down in HL-1 cells by RT-PCR (n = 3 per group); B) Serpine1 expression was decreased after knockdown Serpine1 mRNA in HL-1 cells by Western blot (n = 3 per group). Summary data are presented as the mean±SEM. Statistical signiﬁcance was calculated using one-way ANOVA with Tukey’s multiple comparisons test; ***P* < 0.01.
